# Author Correction: *MiR-31* promotes mammary stem cell expansion and breast tumorigenesis by suppressing Wnt signaling antagonists

**DOI:** 10.1038/s41467-020-19103-2

**Published:** 2020-10-15

**Authors:** Cong Lv, Fengyin Li, Xiang Li, Yuhua Tian, Yue Zhang, Xiaole Sheng, Yongli Song, Qingyong Meng, Shukai Yuan, Liming Luan, Thomas Andl, Xu Feng, Baowei Jiao, Mingang Xu, Maksim V. Plikus, Xing Dai, Christopher Lengner, Wei Cui, Fazheng Ren, Jianwei Shuai, Sarah E. Millar, Zhengquan Yu

**Affiliations:** 1 State Key Laboratories for Agrobiotechnology and Beijing Advanced Innovation Center for Food Nutrition and Human Health, College of Biological Sciences, China Agricultural University, Beijing, 100193 China; 2 Department of Biochemistry and Molecular Biology, Hebei Key Laboratory of Chinese Medicine Research on Cardio-Cerebrovascular Disease, Hebei University of Chinese Medicine, Shijiazhuang, Hebei, 050200 China; 3 Department of Biochemistry and Molecular Biology, Basic Medical College, Tianjin Medical University, Tianjin, 300070 China; 4 Vanderbilt University Medical Center, Nashville, TN 37232 USA; 5 State Key Laboratory of Genetic Resources and Evolution of Kunming Institute of Zoology, Chinese Academy of Sciences, Kunming, Yunnan 650223 China; 6 Department of Dermatology, Perelman School of Medicine, University of Pennsylvania, Philadelphia, PA 19104 USA; 7 Department of Developmental and Cell Biology, Sue and Bill Gross Stem Cell Research, Center for Complex Biological Systems, University of California, Irvine, CA 92697 USA; 8 Departments of Biological Chemistry and Dermatology, School of Medicine, University of California, Irvine, CA 92697 USA; 9 Department of Biomedical Sciences, School of Veterinary Medicine, University of Pennsylvania, Philadelphia, PA 19104 USA; 10 Institute for Regenerative Medicine, University of Pennsylvania, Philadelphia, PA 19104 USA; 11 Department of Cell and Developmental Biology, Perelman School of Medicine, University of Pennsylvania, Philadelphia, PA 19104 USA; 12 Institute of Reproductive and Developmental Biology, Department of Surgery and Cancer, Imperial College London, London, W12 0NN UK; 13 Department of Physics and State Key Laboratory of Cellular Stress Biology, Innovation Center for Cell Signaling Network, , Xiamen University, Xiamen, 361005 China

**Keywords:** Mammary stem cells, Breast cancer

Correction to: *Nature Communications* 10.1038/s41467-017-01059-5, published online 19 October 2017.

The original version of this Article contained an error in Fig. 4. In the original Fig. 4a (shown below), different quadrants within the same FACS plot and between different plots contained similar unexplained groups of data points. The raw data of the original figure are not available as they were deleted at the FACS facility where the analysis was performed. The experiments have several original replicates, for which raw data are available. The experiments have also been repeated for added robustness and the new version of Fig. 4a is shown below. This has been corrected in both the PDF and HTML versions of the Article.

Original version of Fig. 4 

Corrected version of Fig. 4 

The original conclusion of a significant increase in CD24+/CD29-high mammary epithelial cells in DTG vs. control mice (as shown in Fig. 4a) remains unchanged and was already supported by the original Fig. 8i. In addition, repeats, gating strategies and raw data of Fig. 4a as well as gating strategies and raw data of the original Fig. 8i have been deposited in figshare (10.6084/m9.figshare.13013813).

