# Supplementary material for: MiR-31 promotes mammary stem cell expansion and breast tumorigenesis by suppressing Wnt signaling antagonists
Source: Nat Commun. 2017 Oct 19;8:1036. doi: 10.1038/s41467-017-01059-5 (PMC5648844; doi:10.1038/s41467-017-01059-5)
Supplement: Supplementary file 1 — Supplementary Information [file 41467_2017_1059_MOESM1_ESM.pdf]

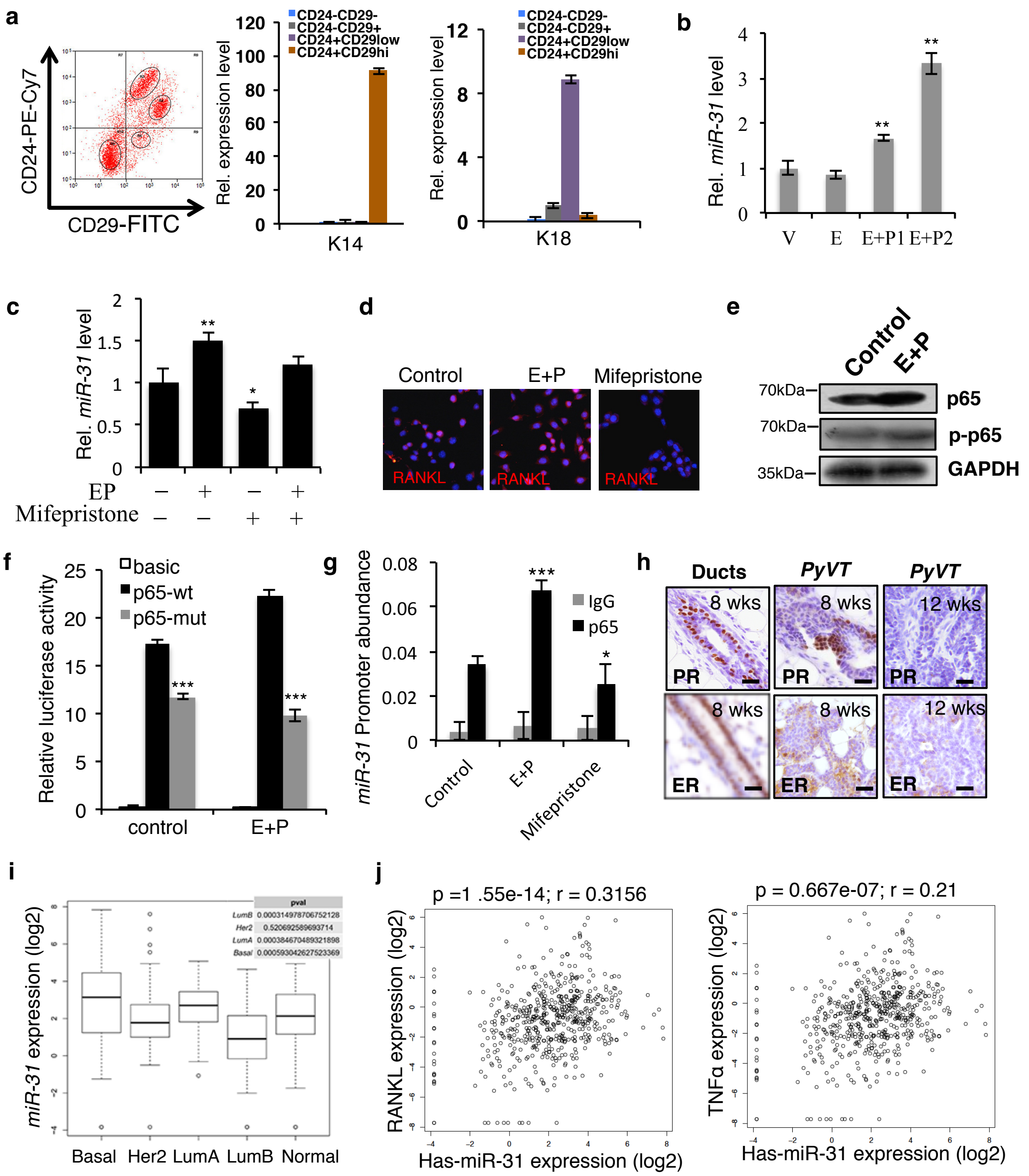

Supplementary Figure 1. *MiR-31* is regulated by progesterone. (**a**) Representative cell sorting profiles using CD24-PE-Cy7 and CD29-FITC in cell suspensions of WT mammary glands. qRT-PCR showing expression of basal marker gene, K14, and of luminal marker gene, K18, in $CD24^{-}CD29^{-}$, $CD24^{-}CD29^{+}$, $CD24^{+}CD29^{low}$ and $CD24^{+}CD29^{high}$ subpopulations. n = 3 biological replicates. (**b**) qRT-PCR analysis for *miR-31* in HC11 mouse mammary epithelial cells under conditions of vehicle, Estradiol, E+P1 (10nM Estradiol and 100 nM progesterone, n = 9 technicalbiological replicates), and E+P2 (10nM Estradiol and 1μM progesterone, n = 4 technicalbiological replicates). (**c**) qRT-PCR analysis for *miR-31* in HC11 mouse mammary epithelial cells treated with E+P

(10nM Estradiol and 100 nM progesterone) and/or Mifepristone. n = 3 technical replicates. (**d**) Immunofluorescence for RANKL in HC11 mouse mammary epithelial cells treated with Mifeprisetone, Estradiol and Progesterone (E+P) or vehicle control. n = 3 technical replicates. (**e**) Western blotting for p65 and p-p65 in HC11 mouse mammary epithelial cells treated with vehicle control or Estradiol and Progesterone (E+P). GAPDH was used as a loading control. n = 3 technical replicates. (**f**) Luciferase activity in lysates of HC11 mammary epithelial cells transfected with luciferase reporter plasmid of pGL3-basic, *miR-31* promoter and *miR-31* mutant promoter with mutation at the -1375 binding site, treated with Vehicle or Estradiol and Progesterone (E+P). n = 3 technical replicates. (**g**) Chromatin immunoprecipitation (ChIP) assay carried out on HC11 mammary epithelial cells using antibodies against p65 under indicated conditions. The enrichment of p65 binding to the site of -1375 bp in *miR-31* promoter was quantified using qPCR. (**h**) Immunohistochemistry for ER and PR in *PyVT* tumors and mammary ducts at 8 and/or 12 weeks of age. Scale bar: 50 μm. n = 3 biological replicates. (**i**) Expression of *miR-31* across different breast tumors subtypes in TCGA RNA-Seq. (**j**) Pearson correlation analysis on *miR-31* and RANKL (P value = 1.55e-14; r = 0.3156), as well as *miR-31* and TNFα (P value = 0.667e-07; r = 0.21) in breast cancer TCGA RNA-seq. Data represented as mean ±S.D. Two tailed unpaired *t*-test for b, c, f, g (*$P < 0.05$; **$P < 0.01$; ***$P < 0.001$).

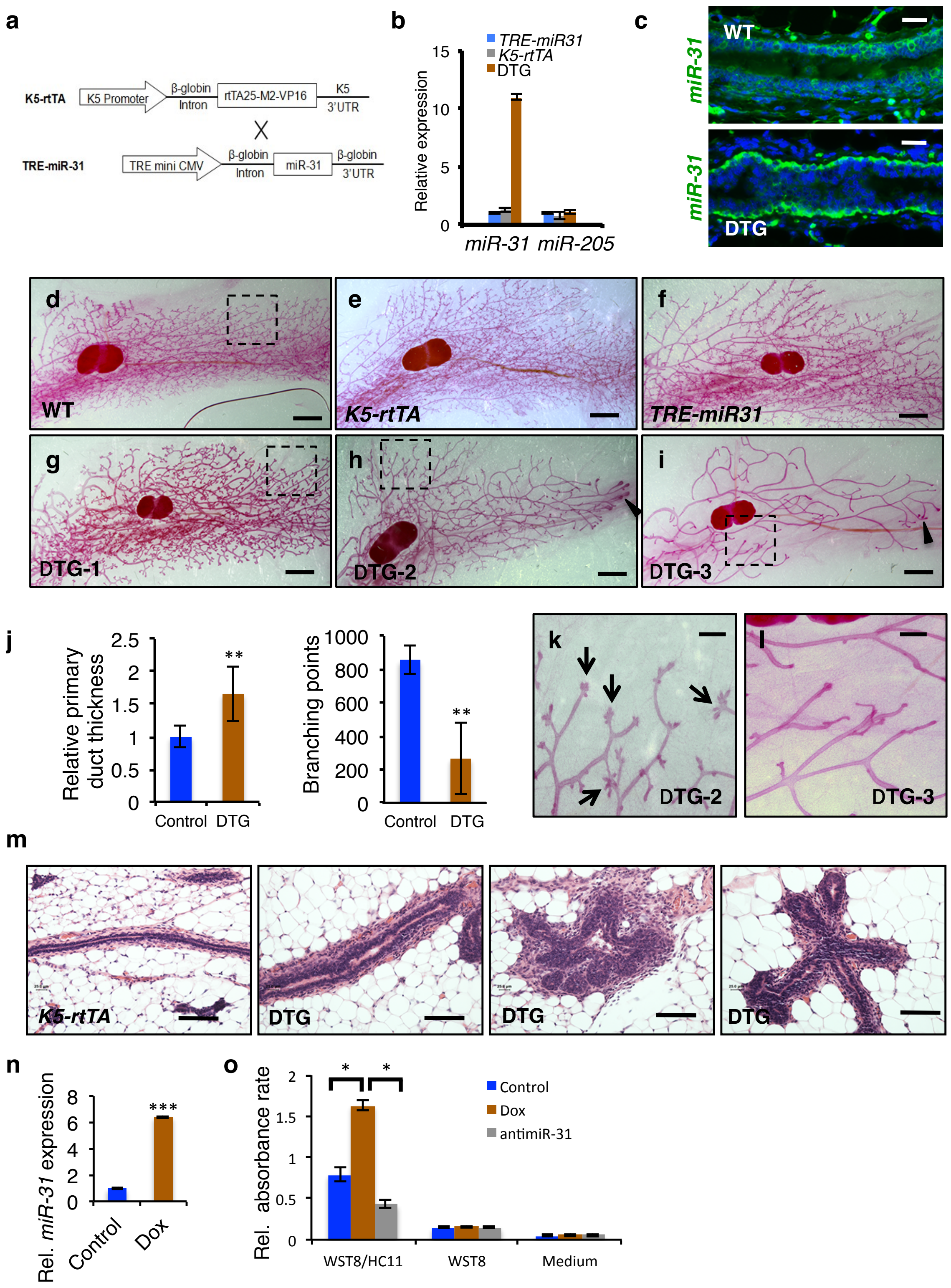

Supplementary Figure 2. *MiR-31* induction causes impaired branching and mammary epithelial hyperplasia. (**a**) Schematic maps of constructs used to generate *K5-rtTA/TRE-miR31* (DTG) double transgenic mice. (**b**) qRT-PCR for *miR-31* and *miR-205* in $CD24^{+}CD29^{high}$ cells of *TRE-miR31*, *K5-rtTA* and DTG

transgenic mammary glands at 12 weeks of age following Dox treatment from 1 week of age. n = 3 technical replicates. (**c**) *In situ* Hybridization for *miR-31* in WT and DTG mammary ducts at 12 weeks of age following Dox treatment from 1 week of age. (**d-i**) Whole-mount staining of WT (n = 3), *K5-rtTA* (n = 3), *TRE-miR31* (n = 3) and DTG mammary glands at 12 weeks of age. DTG-1(**g,** n = 3), DTG-2 (**h,** n = 3) and DTG-3 (**I,** n = 3) represent 3 classes of DTG mammary gland phenotypes. The areas outlined by dashed boxes in panel **d** and **g** are shown at higher magnification in main Fig. 2a. Those dashes boxes in panels **h** and **i** are shown in **k** and **l**, respectively. Arrowheads indicate TEBs. Scale bars: 1mm. (**j**) Quantification of primary duct thickness (Left panel) and mammary branch points (Right panel) in Control (n = 3) and DTG (n = 3) mice at 12 weeks of age. Y axis in Left panel represents fold change of diameter in primary duct. (**k,l**) The higher images indicated by dashed boxes in (**h**) and (**i**). Scale bar: 0.2 mm. (**m**) H&E staining showing histology of *K5-rtTA* (n = 3) and DTG (n = 3) mammary glands at 12 weeks of age following Dox treatment at 1 week of age. Scale bar: 50 μm. (**n**) qRT-PCR analysis for *miR-31* in *miR-31* inducible HC11 mammary epithelial cells following treatment of Dox. n = 3 technical replicates. (**o**) WST-8 cell proliferation assay showing that *miR-31* overexpression promotes cell proliferation, whereas *anti-miR-31* has no significant effect. n = 3 technical replicates. Data represented as mean ± S.D. n = 3. Two tailed unpaired *t*-test for j, n, o (*$P < 0.05$; **$P < 0.01$; ***$P < 0.001$).

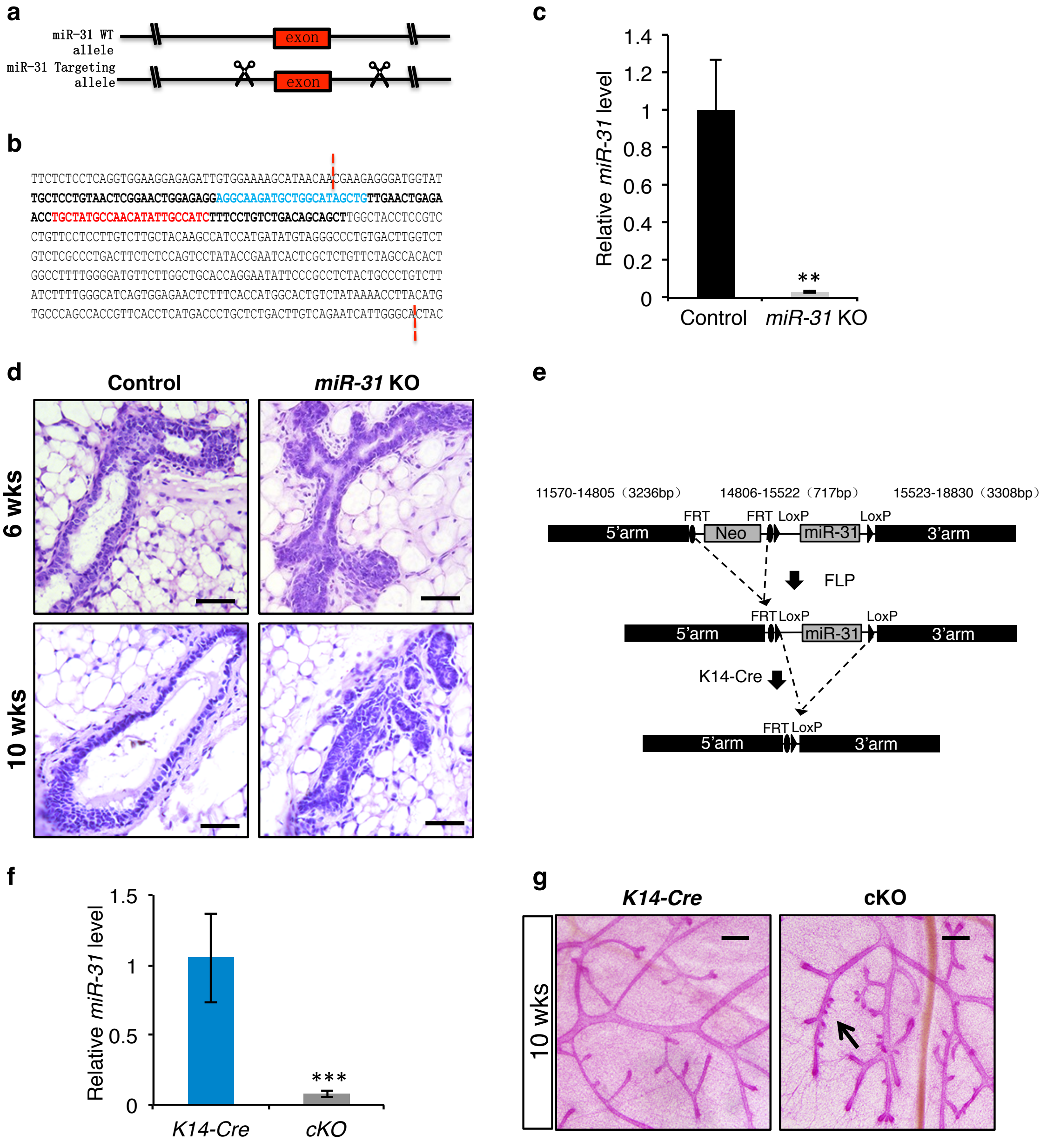

Supplemental Figure 3. Generation of constitutive *miR-31* null and a conditional *miR-31* null allele. (**a**) Strategy to generate *miR-31* KO mice using CRISPR/Cas9 RNA-guided nucleases. (**b**) The 402 bp DNA fragment containing *miR-31* indicated by dash lines was deleted in the KO allele. The *miR-31* exon indicated by bold; mature *miR-31* indicated by blue, *miR-31** indicated by red. (**c**) qRT-PCR analysis for *miR-31* in Control (n = 3) and *miR-31* KO (n = 3) mice. (**d**) H&E staining of Control (n = 3) and *miR-31* KO (n = 3) mammary gland at 6 weeks and 10 weeks of age. Scale bar: 50 μm. (**e**) Strategy to generate *miR-31* conditional KO allele in mammary epithelium. (**f**) qRT-PCR analysis for *miR-31* in

K14-Cre (n = 3) and cKO (*K14-Cre*;*miR-31*$^{fl/fl}$, n = 3) mice. (**g**) Whole-mounts of mammary gland from *K14-Cre* (n = 3) and cKO (n = 3) mice at 10 weeks of age. Scale bar: 0.2 mm. Data represented as mean ± S.D. n = 3. Two tailed unpaired *t*-test for c, f (***P* < 0.01; ****P* < 0.001). Note: The schematic depiction of the general strategy for generating *miR-31* mutant mice **(Supplementary Figure 3a and b)** was also used in another unrelated study on the role of *miR-31* in intestinal stem cells and colorectal cancer, and the manuscript is currently accepted in *eLife*.

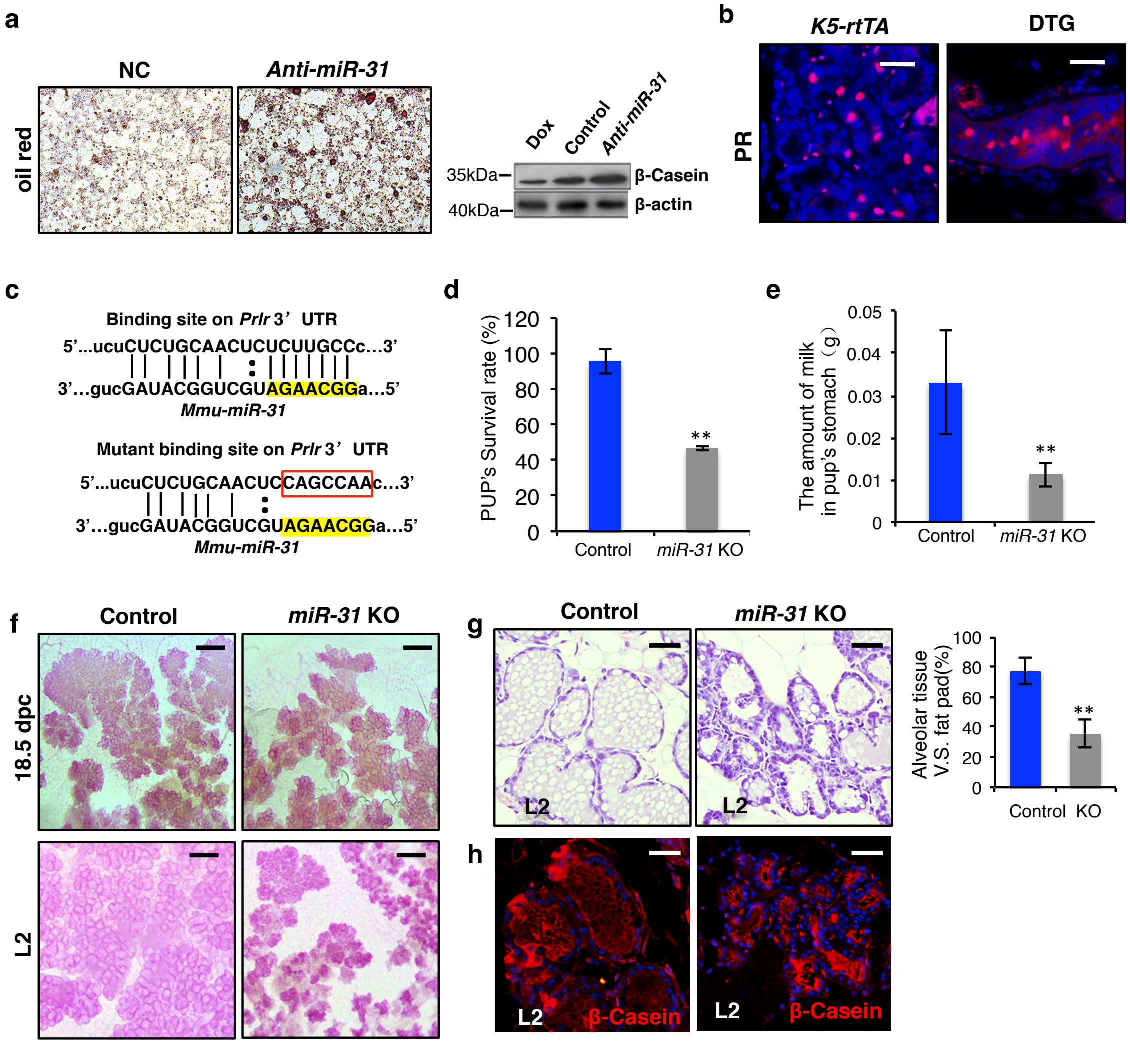

Supplementary Figure 4. *MiR-31* is important for normal alveogenesis during pregnancy. (**a**) Red Oil staining in HC11 mouse mammary epithelial cells infected with scramble RNA (NC) and *anti-miR-31* after lactogenic hormone-induced cell differentiation (Left panel). Western blot analysis showing reduced β-casein protein levels in *miR-31* overexpressing HC11 cells (Dox induced *miR-31* over-expression) and increased β-casein protein in *anti-miR-31* infected HC11 cells after lactogenic hormone-induced cell differentiation (Right panel). β-actin was used as a loading control. (**b**) Immunofluorescence for PR in *K5-rtTA* (n = 3) and DTG (n = 3) mammary glands at 14.5 dpc following Dox treatment at 3 weeks of age. Scale bar: 25 µm. (**c**) Top panel indicates *miR-31* binding site in wild type *Prlr* 3'UTR regions. Bottom panel depicts mutant *miR-31* binding site. Seed sequences are marked by yellow. Red box indicates the mutated *miR-31* binding sites in the *Prlr* 3'UTR. (**d**) Statistical analysis of survival rate of pups raised by WT (Control, n = 3) and *miR-31* KO (n = 3) mothers at weaning age.

KO, n = 21; WT, n = 22. (**e**) Quantification of milk amount from pup's stomach at lactation day 2, in which pups raised by WT (Control, n = 14) and *miR-31* KO (n = 12) mothers. (**f**) Whole mount analysis for Control (n = 3) and *miR-31* KO (n = 3) mammary glands at 18.5 dpc and lactation day 2 (L2). Scale bar: 0.2 mm. (**g**) H&E staining for Control (n = 3) and *miR-31* KO (n = 3) mammary gland at lactation day 2 (L2). Statistics of proportion of mammary alveolar tissue v.s. fat pad. Scale bar: 25 μm. (**h**) Immunofluorescence for β-Casein in Control (n = 3) and *miR-31* KO (n = 3) mammary glands at lactation day 2 (L2). Scale bar: 25 μm. Data represented as mean ± S.D. $n \geq 3$. Two tailed unpaired *t*-test for d, e, g (**$P < 0.01$).

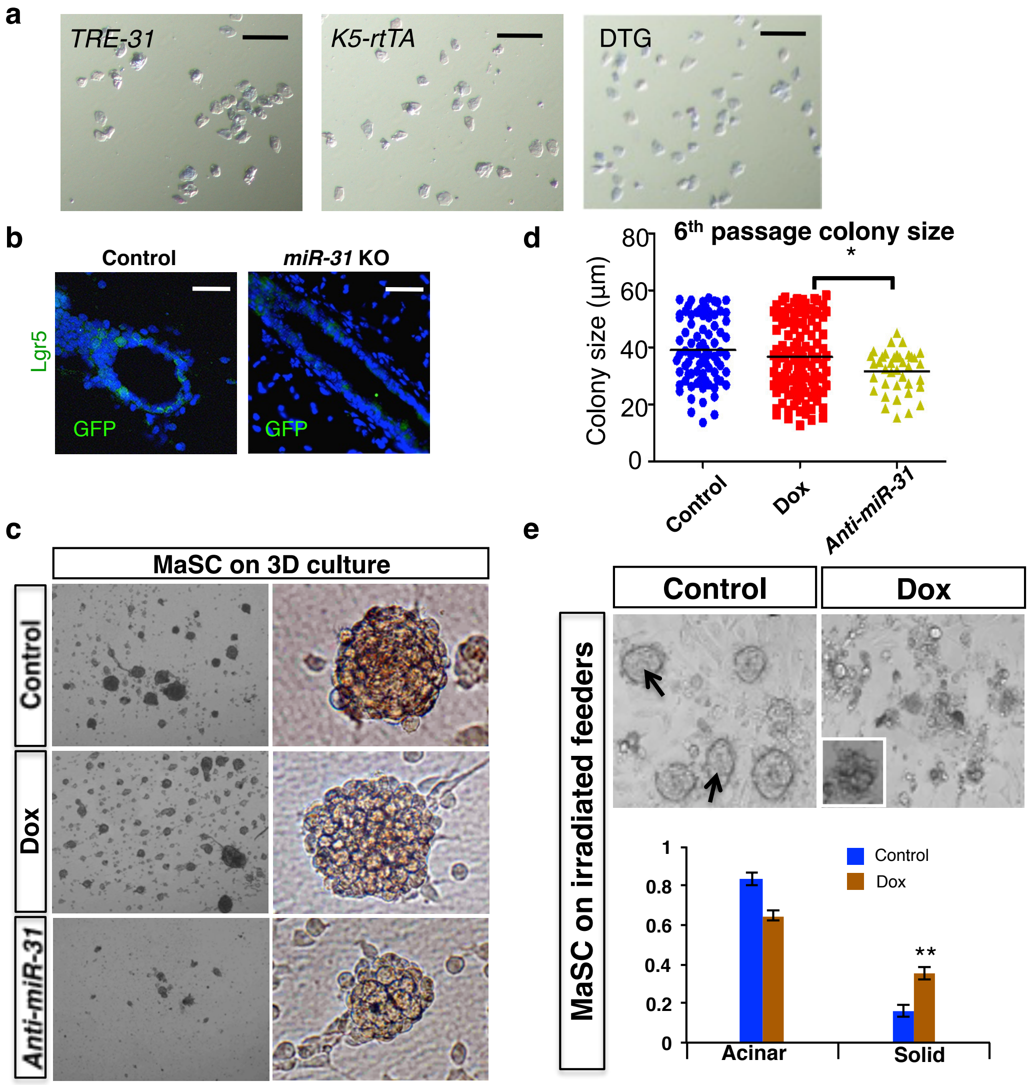

Supplementary Figure 5. *MiR-31* induces mammary basal stem cell expansion. (**a**) Images of vaginal smears taken from *TRE-miR31*, *K5-rtTA* and DTG mice at proestrus phase, related to Fig. 4a. (**b**) GFP immunofluorescence in mammary ducts from *Lgr5-eGFP-CreER* (Control, n = 3) and *Lgr5-eGFP-CreER*;*miR-31* KO (*miR-31* KO, n = 3) mice. Scale bar: 50 µm. (**c**) Representative images of colonies formed on culture inserts under conditions of Control (Scramble RNA), Dox treatment (Dox induced *miR-31* overexpression) and *anti-miR-31* transfection. High magnification images of single colonies are shown in the right panels. (**d**) Colony sizes at 6th passage under the indicated conditions. n = 3 technical replicates. (**e**) Representative images of colonies formed on irradiated feeder layers in the presence or absence of Dox. Arrows indicate acinar colonies. Inset shows a solid colony. The graph shows statistical results of solid colony and acinar colony formation in the presence or absence of Dox. Data represented as mean ± S.D. n = 3. Two tailed unpaired *t*-test for d, e (*$P < 0.05$; **$P < 0.01$).

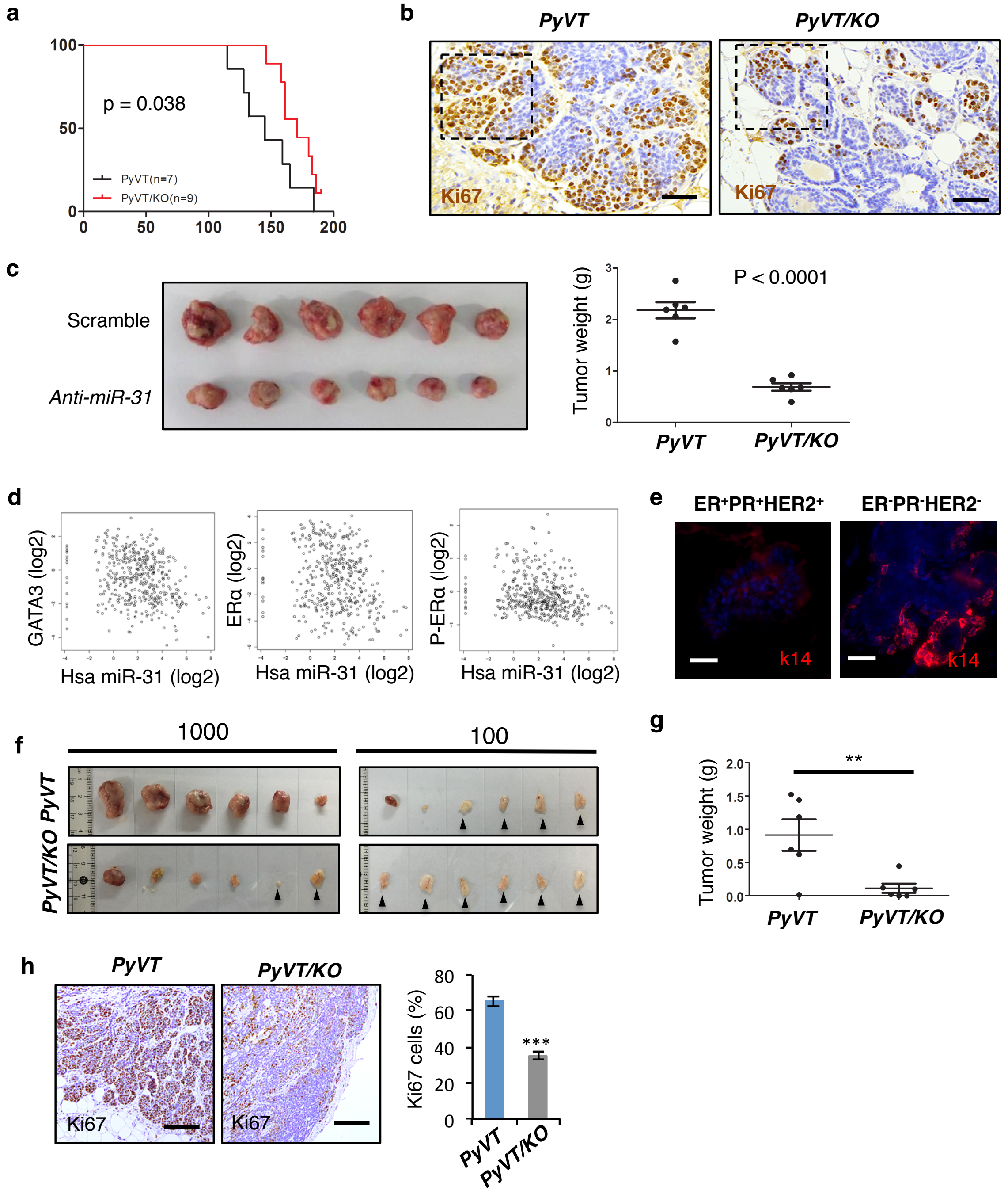

Supplementary Figure 6. Loss of *miR-31* resulted in compromised tumor growth, reduced cancer stem cells and impaired lung metastasis. (**a**) The statistical analysis on overall survival rate of *PyVT* (n = 7) and *PyVT/KO* (n = 9) mice. (**b**) Immunohistochemistry for Ki67 in *PyVT* (n = 3) and *PyVT/KO* (n = 3) tumors at 12 weeks of age. The higher magnification images indicated by dashed boxes were shown in Fig. 5d. Scale bar: 50 μm. (**c**) Gross appearance of engrafted tumors with 5 x 10^4 4T1 (n= 6 mice) and *anti-miR-31*-treated 4T1 (n = 6 mice) mouse breast cancer cells engrafted 4 weeks post transplantation.

Quantification of tumor weight of the engrafted tumors. (**d**) Pearson correlation analysis on *miR-31* and GATA3 (P = 8.99e-08, r = -0.26), ERα (P = 1.74e-06, r = -0.23), p-ERα (P = 1.88e-05, r = -0.21) in TCGA breast cancer RNA-Seq, respectively. (**e**) Immunofluorescence for K14 in $ER^+PR^+HER2^+$ and $ER^-PR^-HER^-$ human breast tumors. Scale bar: 25 μm. (**f**) Gross appearance of tumors transplanted with 1000 (n = 6 mice) and 100 (n = 6 mice) of *PyVT* and *PyVT/KO* tumor cells, engrafted 8 weeks post transplantation. Arrowheads point to fat pads (not tumors). Related to Fig. 5j. (**g**) Quantification of tumor weight of the engrafted tumors transplanted with 1000 *PyVT* and *PyVT/KO* tumor cells in Panel **f**. (**h**) Immunohistochemistry for Ki67 and quantification of Ki67 positive cells in the xenografted tumors transplanted with 1000 *PyVT* and *PyVT/KO* tumor cells in Panel **f**. Scale bar: 100 μm. n = 3 biological replicates. Data represented as mean ± S.D. n ≥ 3. Two tailed unpaired *t*-test for c, g, h (*$P < 0.05$; **$P < 0.01$; ***$P < 0.001$). Survival curves were estimated by the Kaplan-Meier method and compared using the Wilcoxon test for panel **a**.

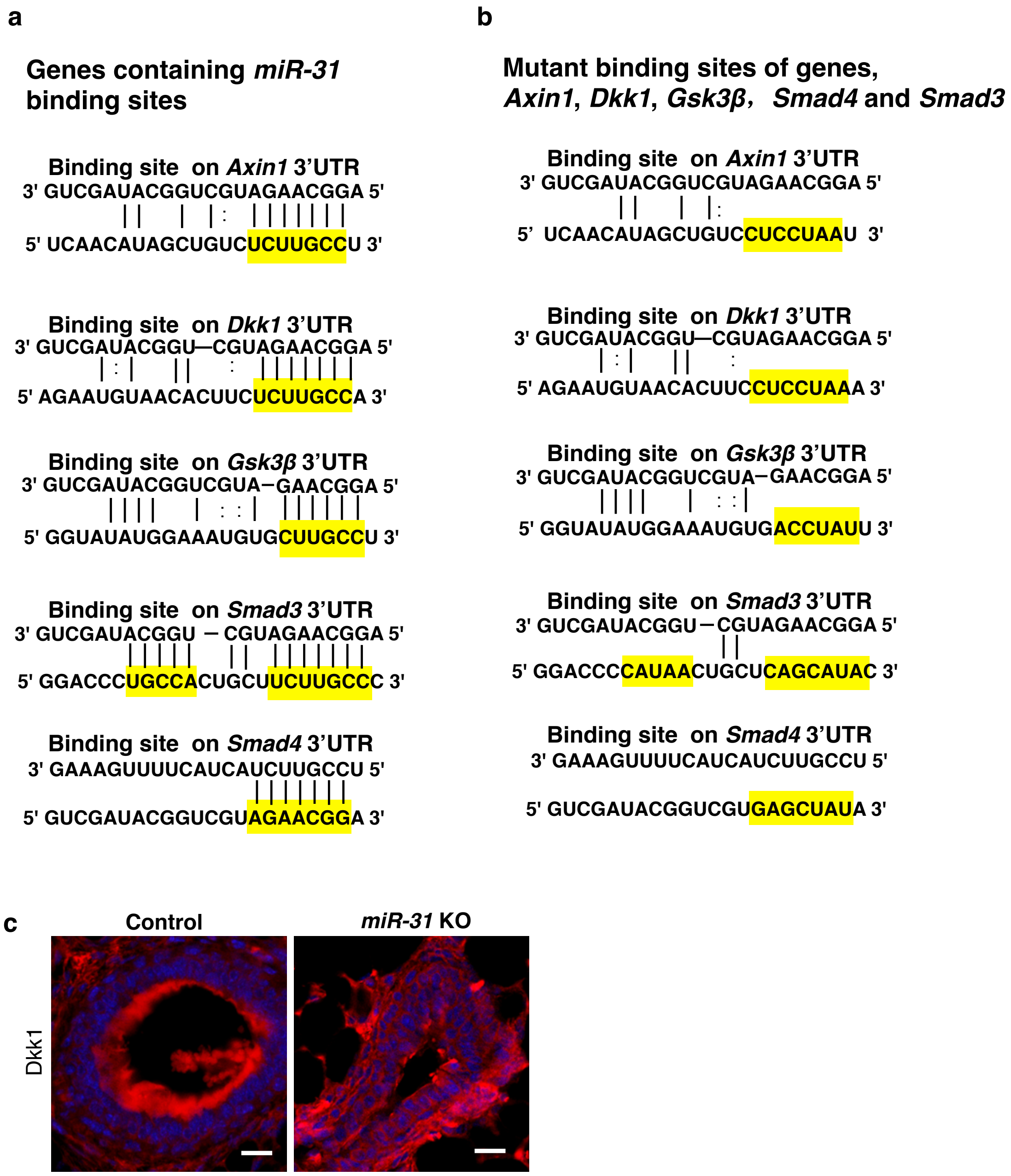

Supplementary Figure 7. Identification of *miR-31* binding sites. (**a**) 3'UTR of *Axin1, Dkk1*, *Gsk3β*, *Smad4* and *Smad3* containing *miR-31* binding sites. Yellow indicated *miR-31* seed sequence. (**b**) Mutant binding sites of genes, *Axin1*, *Dkk1*, *Gsk3β*, *Smad4* and *Smad3*. Yellow indicated *miR-31* mutant seed sequence. (**c**) Immunofluorescence for Dkk1 in Control (n = 3) and *miR-31* KO (n = 3) mammary ducts. Scale bar: 25 μm.

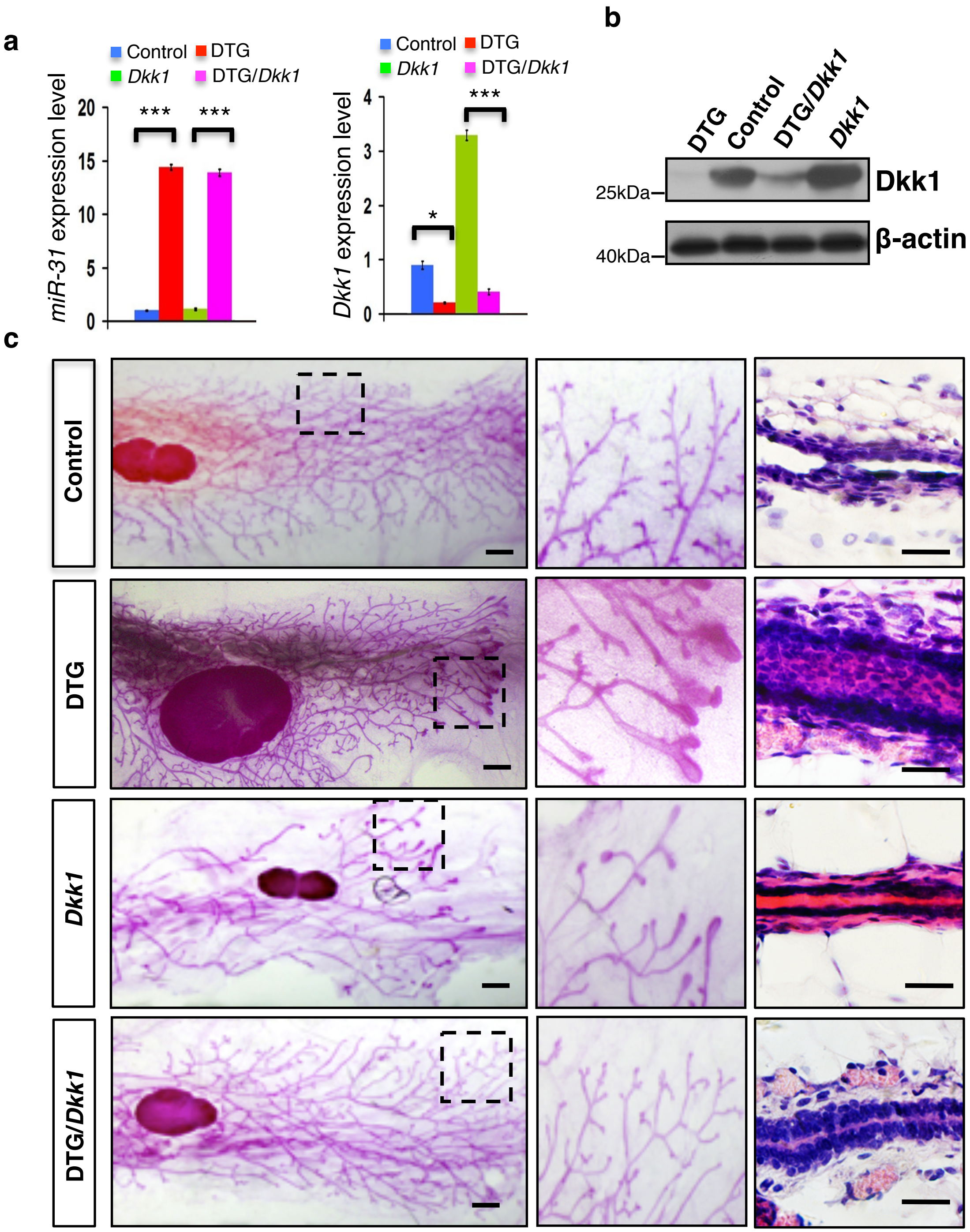

Supplementary Figure 8. *Dkk1* induction can partially rescue DTG mammary phenotype. (**a**,**b**) qRT-PCR for *miR-31* and *Dkk1* (**a**), and Western blotting for Dkk1 (**b**) in Control (*K5-rtTA*), DTG (*K5-rtTA;TRE-miR31*), *Dkk1* (*K5-rtTA*;*TRE-Dkk1*), and DTG/*Dkk1* (*K5-rtTA;TRE-miR31;TRE-Dkk1*) mammary glands at 12 weeks of age following Dox induction at 1 week of age. β-actin was used as a loading control. (**c**) Whole-mount staining of mammary glands from *K5-rtTA*, DTG, *Dkk1* and DTG/*Dkk1* mice at 12 weeks of age following Dox induction

at 1 week of age. Regions outlined by dashed boxes are shown at higher magnification in the respective middle panels. Histology of the corresponding mammary ducts is shown in the right panels. 3 *K5-rtTA*, 3 DTG, 3 *Dkk1* and 3 DTG/*Dkk1* mice were analyzed. Scale bar: Left panels, 1 mm; Right panels: 25 μm. Data represented as mean ±S.D. n = 3. Two tailed unpaired *t*-test for a (*$P <$ 0.05; ***$P <$ 0.001).

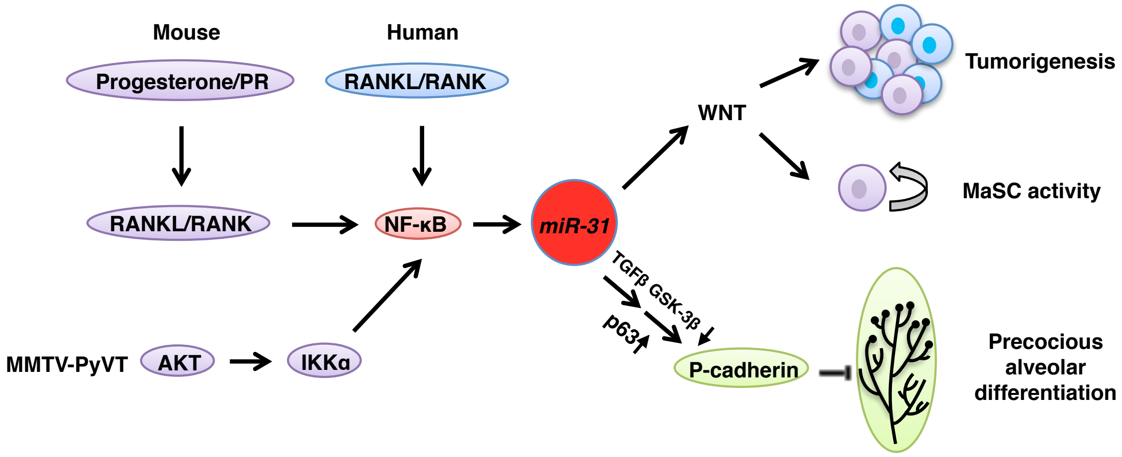

Supplementary Figure 9. The working model of *miR-31* in regulating mammary gland development, MaSC activity and breast tumorigenesis.

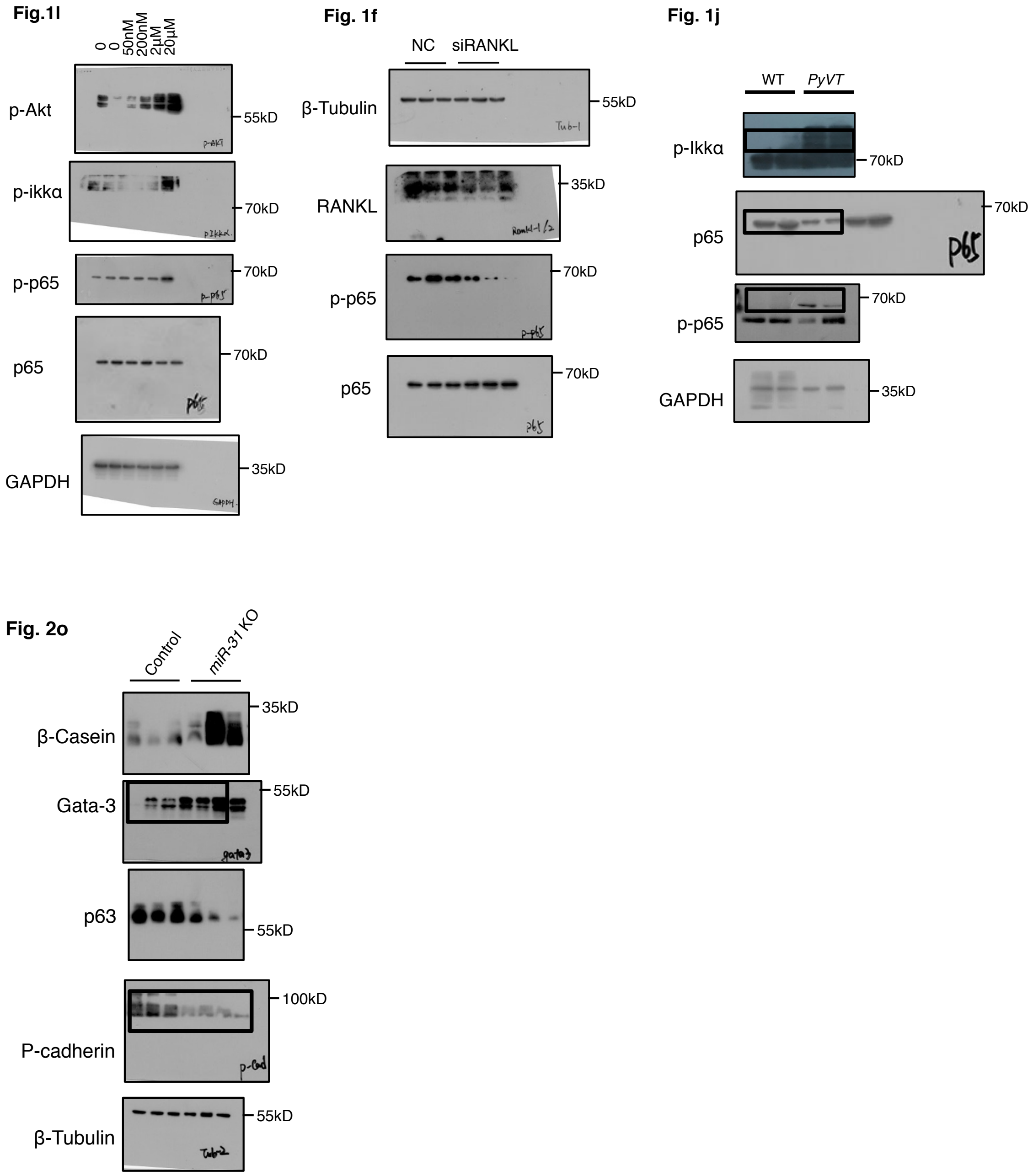

Supplementary Figure 10. Uncropped blots for western blot analysis in the main figures.

Fig. 3g

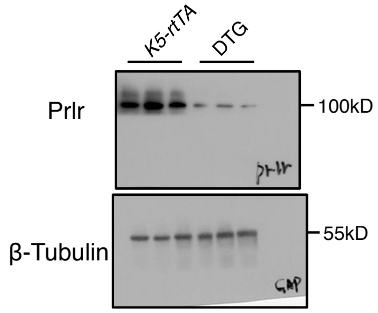

Fig. 3l

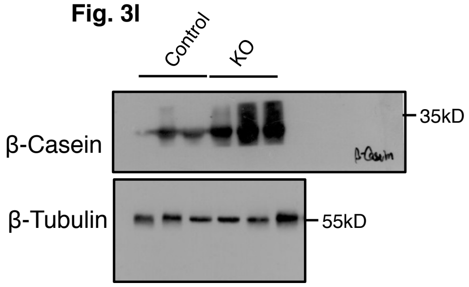

Fig. 3o

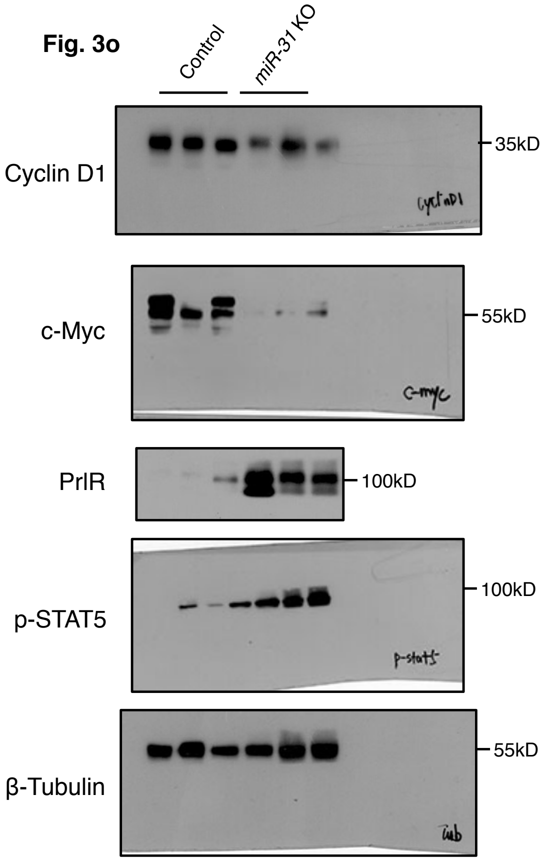

Fig. 5f

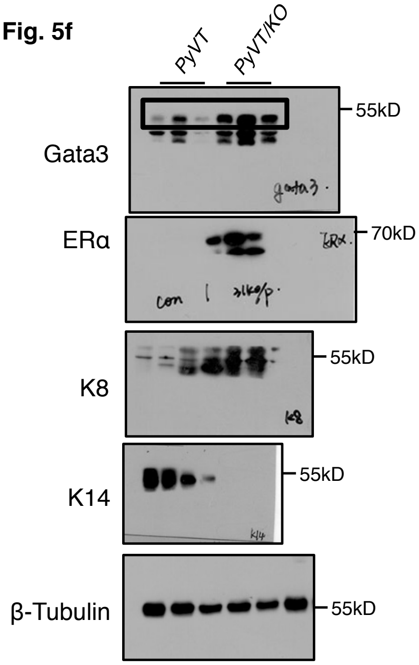

Fig. 6h

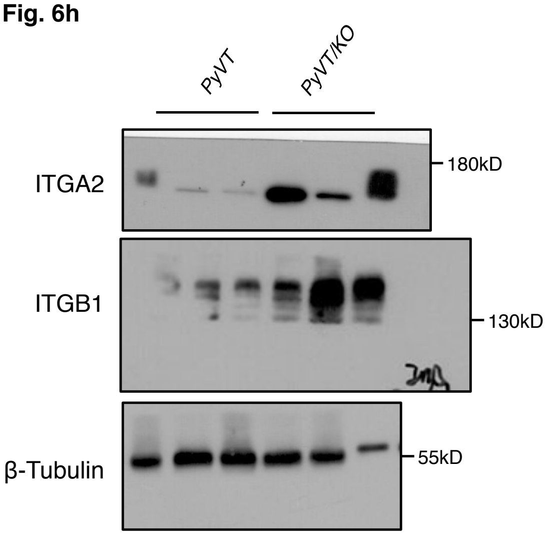

Supplementary Fig. 10 continued

Fig. 7c

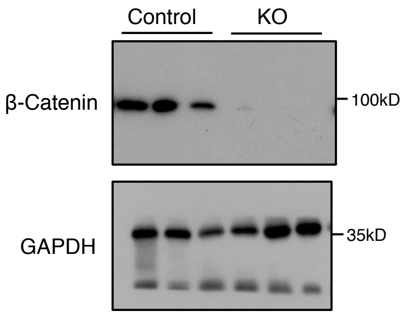

Fig. 7g

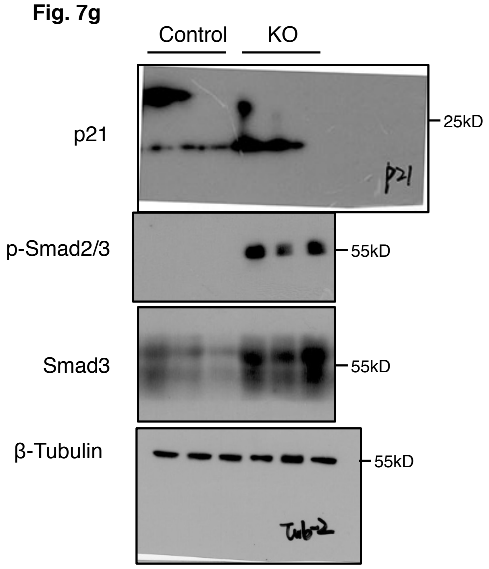

Fig. 8c

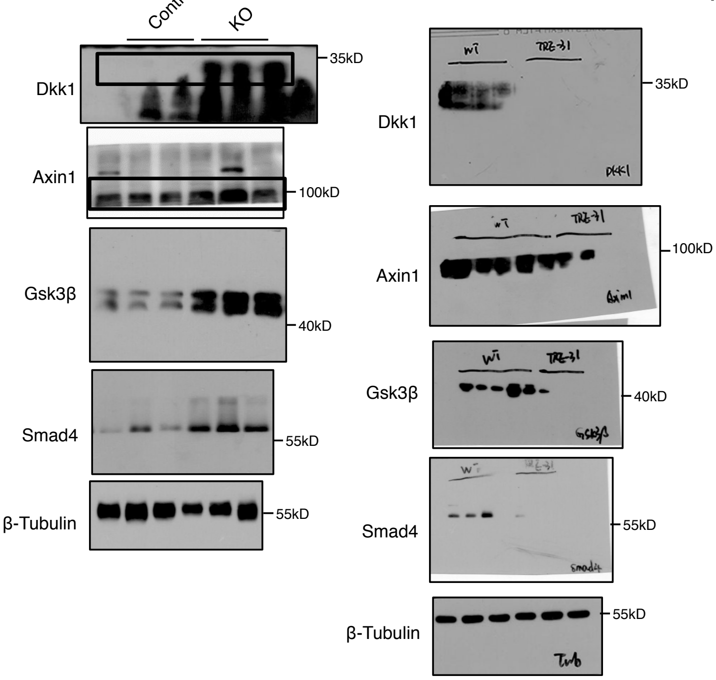

Fig. 8g

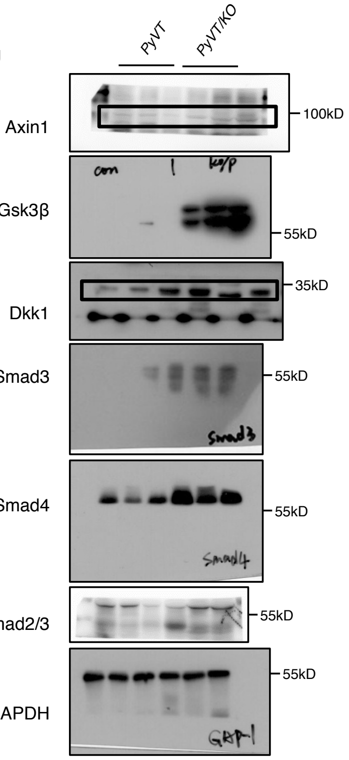

Supplementary Fig. 10 continued
